# Home Fetal Heart Rate Monitoring in Pregnancy: Patient Experience and Acceptance in the Era of Digital Prenatal Care

**DOI:** 10.3390/healthcare14121702

**Published:** 2026-06-15

**Authors:** Sidonia Maria Săndulescu, Virginia Maria Rădulescu, Sidonia Cătălina Vrabie, Anca Vulcănescu, Andreea Velișcu Carp, Mirela Anișoara Siminel, George Lucian Zorilă, Ioana Victoria Camen, Laurențiu Dîră, Bogdan Ivănuș, Claudia Monica Danilescu, Maria-Magdalena Manolea

**Affiliations:** 1 “Filantropia” Clinical Municipal Hospital, 200143 Craiova, Romania; sidonia.sandulescu@umfcv.ro (S.M.S.); mirela.siminel@umfcv.ro (M.A.S.); ci_victoria0701@yahoo.com (I.V.C.); laurentiu.dira@yahoo.com (L.D.); bogdanliv@yahoo.com (B.I.); magdalena.manolea@umfcv.ro (M.-M.M.); 2 Department of Obstetrics and Gynecology, University of Medicine and Pharmacy of Craiova, 200349 Craiova, Romania; zorilalucian@gmail.com; 3 Department of Medical Informatics and Biostatistics, University of Medicine and Pharmacy of Craiova, 200349 Craiova, Romania; virginia.radulescu@umfcv.ro; 4 Faculty of Medicine, University of Medicine and Pharmacy of Craiova, 200349 Craiova, Romania; 5 Department of Obstetrics Gynecology, Carol Davila University of Medicine and Pharmacy, 050474 Bucharest, Romania; andreea_veliscu@yahoo.com; 6 Panait Sirbu Hospital, 060251 Bucharest, Romania; 7 Department of Neonatology, University of Medicine and Pharmacy of Craiova, 200349 Craiova, Romania; 8 Clinical Emergency County Hospital, 200642 Craiova, Romania; 9 Department of Home Care and Community Nursing, University of Medicine and Pharmacy of Craiova, 200349 Craiova, Romania; monica.danilescu@umfcv.ro

**Keywords:** fetal heart rate monitoring, home monitoring, pregnancy, prenatal care, patient experience, telemedicine, digital health

## Abstract

**Background**: Digital health technologies have expanded access to home fetal heart rate (FHR) monitoring devices, enabling fetal surveillance outside clinical settings. However, evidence on women’s awareness, acceptance, and experiences with these devices remains limited. **Objective**: To assess awareness, adoption, user experience, perceived reassurance, and attitudes toward home FHR monitoring among pregnant and postpartum women. **Methods**: A cross-sectional online survey was conducted using a structured questionnaire distributed via Google Forms. Eligible participants were women aged ≥18 years who were currently pregnant or had been pregnant within the previous two years. The survey evaluated awareness and use of home FHR monitoring devices, usage patterns, sources of recommendation and instruction, emotional responses, perceived reassurance, mobile application integration, and overall attitudes. Descriptive statistics and exploratory subgroup analyses were performed. **Results**: A total of 225 women completed the survey; 166 (73.8%) reported using a home FHR monitoring device during pregnancy. Most users reported positive emotional experiences, with calmness as the most common response. Home monitoring was generally perceived as reassuring, and many participants felt calmer on days of device use. Gynecologists were the primary source of device recommendations and usage instructions. Participants highlighted the importance of professional guidance, clear instructions, and mobile application support. Primiparous women had significantly higher adoption rates than multiparous women (*p* < 0.001). **Conclusions**: Home FHR monitoring was widely accepted and commonly perceived as reassuring. These devices may support patient-centered prenatal care when accompanied by appropriate professional guidance. Further prospective studies are needed to assess their clinical utility, safety, and integration into prenatal care pathways.

## 1. Introduction

Despite substantial advances in obstetric care and prenatal diagnostic technologies, fetal distress remains a major contributor to perinatal morbidity and mortality worldwide. Fetal compromise is often associated with intrauterine hypoxia and placental insufficiency—conditions that can be difficult to detect early and for which standardized management approaches are not always clearly established. These conditions are frequently related to maternal vascular disorders and may lead to adverse outcomes such as preterm birth or fetal death. Importantly, fetal deterioration may occur even in the absence of obvious clinical signs, making early recognition particularly challenging. These limitations highlight the need for more effective and accessible monitoring approaches capable of supporting earlier recognition of concerning changes during pregnancy [1].

Fetal heart rate (FHR) monitoring represents a cornerstone of modern obstetric practice, providing essential information on fetal well-being during both pregnancy and labor. Monitoring strategies vary according to clinical risk, ranging from intermittent assessment in low-risk pregnancies to continuous surveillance in high-risk settings. Conventional methods include Doppler ultrasound and fetal electrocardiography, typically integrated into cardiotocography systems [2]. Abnormal findings, such as baseline heart rate abnormalities, reduced variability, or pathological decelerations, guide clinical decision-making and obstetric intervention [3].

However, conventional hospital-based monitoring systems present several limitations. Signal quality may be influenced by maternal body habitus, fetal position, and movement, leading to intermittent signal loss during routine monitoring [4]. Although internal monitoring techniques provide more stable recordings, their invasive nature limits their use to selected clinical scenarios [5]. These challenges have encouraged the advancement of more adaptable monitoring technologies that aim to improve both data accuracy and user comfort.

Maternal positioning during fetal monitoring may influence uteroplacental perfusion and signal quality; therefore, approaches that allow greater mobility and flexibility, including wireless monitoring systems, represent an important advancement in improving both patient comfort and monitoring reliability [6,7,8,9].

Recent advances include wireless and non-invasive systems based on fetal electrocardiography and uterine electromyography, which enable continuous monitoring with reduced dependence on maternal position and improved mobility [6,7,8]. In parallel, the rapid expansion of digital health and telemonitoring platforms has facilitated new models of care, particularly in high-risk pregnancies, where remote monitoring has shown promising feasibility and safety profiles [9,10].

In low-resource settings, innovative monitoring solutions have been developed to address limited access to obstetric care. Wireless fetal monitoring devices have demonstrated good functionality and acceptability among both patients and healthcare providers, with potential to expand access to fetal surveillance and support communication with healthcare providers [11,12]. At the same time, advances in wearable and sensor-based technologies have enabled continuous maternal–fetal monitoring across diverse clinical environments [8].

More recently, the availability of portable monitoring technologies has extended fetal surveillance beyond the hospital setting. Home-based FHR monitoring devices have emerged as a potential complementary approach to prenatal care, particularly in low-risk pregnancies. Studies have shown good agreement between these systems and standard CTG, suggesting that reliable monitoring may be achievable outside clinical settings [13]. However, their broader implementation remains limited by concerns regarding signal accuracy, data interpretation, and appropriate use in the absence of direct medical supervision [14].

In this context, patient-centered factors—including usability, acceptance, and confidence in device use—play a key role in the successful integration of these technologies into routine care. Beyond technical performance, understanding how pregnant women perceive and engage with home monitoring devices is essential for the development of effective and scalable digital health strategies in prenatal care.

Accordingly, the present study aimed to evaluate pregnant women’s awareness, experience, and acceptance of home fetal heart rate monitoring devices, and to explore their potential contribution to patient-centered and digitally supported prenatal care.

## 2. Methods

### 2.1. Study Design

This study was designed as an exploratory, cross-sectional observational survey focusing on patient-reported perceptions, experiences, and behaviors related to home fetal heart rate monitoring. The study aimed to capture real-world user perspectives in a non-controlled setting. It was not intended to evaluate clinical outcomes, diagnostic accuracy, or device performance, but rather to assess acceptability, usage patterns, and perceived impact from the patient’s perspective.

### 2.2. Study Population and Eligibility Criteria

Participants were recruited using a convenience sampling strategy. The survey link was distributed electronically through obstetric and gynecological outpatient settings, pregnancy-related online communities, and social media channels accessible to the target population. Participation was voluntary and based on self-selection. No random sampling procedures were applied, and no incentives were offered for participation. The recruitment approach was chosen to facilitate access to women with current or recent pregnancy experience and to obtain exploratory real-world data regarding awareness, use, and perceptions of home fetal heart rate monitoring devices. Consequently, the study sample may not be fully representative of the general pregnant population, and the findings should be interpreted within the context of a convenience sample.

The target population consisted of adult women with current or recent pregnancy experience. Eligible participants were women aged 18 years or older who were either currently pregnant, in the postpartum period, or had experienced at least one pregnancy within the preceding two years. This inclusion framework was designed to capture both contemporaneous and recent retrospective experiences with home fetal heart rate monitoring, thereby providing a broader assessment of device awareness, use, acceptance, and perceived impact across the perinatal period while limiting recall bias. Participants were included if they were able to understand the questionnaire language and provided sufficiently complete responses to allow interpretation of the primary variables. Responses with substantial missing data affecting key variables were excluded. Potential duplicate entries were reviewed manually based on response patterns and timing and were retained only when considered to represent distinct individuals. Because the survey was anonymous and questionnaire-based, eligibility criteria were limited to self-reported information. No clinically verified inclusion or exclusion criteria, and no pregnancy outcomes, were collected or analyzed as part of the study design.

### 2.3. Questionnaire Development and Structure

The questionnaire was an original structured instrument developed specifically for this study. Item generation was informed by existing literature on digital maternal health, home monitoring technologies, and patient-reported experience, as well as by the clinical expertise of the research team.

The instrument was designed to explore multiple domains relevant to real-world use of home fetal monitoring, including:

1. sociodemographic and obstetric background;
2. awareness of home fetal monitoring;
3. device use and timing of adoption;
4. frequency and context of use;
5. sources of medical recommendation and instruction;
6. emotional experience and perceived reassurance;
7. perceived utility and recommendation behavior;
8. perceived concerns; and
9. attitudes toward digital and mobile application integration.

Most items were categorical or ordinal (yes/no, multiple choice, Likert-type scales), complemented by selected open-ended questions to capture qualitative impressions.

The questionnaire was specifically designed to assess subjective perceptions and user experience, and was not intended to evaluate clinical effectiveness, diagnostic performance, or safety outcomes. Content clarity and wording were reviewed by clinicians prior to dissemination. In addition, selected item groupings were explored through preliminary psychometric analysis (e.g., internal consistency) as part of an initial validation approach.

### 2.4. Data Collection and Data Management

Data were collected through an anonymous online questionnaire hosted on Google Forms (Google LLC, Mountain View, CA, USA). The survey was distributed electronically through channels appropriate for reaching the target population. Participation was voluntary, and no incentives were offered.

Following survey closure, responses were exported into Microsoft Excel for data cleaning, coding, and preliminary organization. The dataset was screened for completeness, consistency, and potential duplicate entries. Missing data were retained as such and excluded from item-level analyses without imputation. The cleaned dataset was subsequently imported into IBM SPSS Statistics, Version 26.0 (IBM Corp., Armonk, NY, USA), for statistical analysis.

Only completed or near-complete questionnaires that allowed interpretation of the primary study variables were included in the final analysis.

### 2.5. Study Variables

The main variables of interest were grouped into four categories. Background variables included age group, pregnancy history, parity, and current obstetric status.

Utilization-related variables included awareness of home monitoring devices, prior use, timing of adoption, perceived optimal gestational use, frequency of monitoring, and prior use in previous pregnancies. Participants were categorized as “users” or “non-users” based on self-reported experience.

Experience-related variables included emotional responses during use, perceived reassurance, perceived impact on maternal well-being, clarity of instructions, need for additional guidance, and perception of digital application support.

Attitudinal variables included willingness to recommend device use, perceived benefits and disadvantages, and general concerns.

All variables were interpreted as subjective perceptions and behavioral indicators, and not as objective measures of clinical effectiveness or fetal well-being.

### 2.6. Statistical Analysis

Statistical analysis was performed using IBM SPSS Statistics, Version 26.0 (IBM Corp., Armonk, NY, USA). Categorical variables were summarized as frequencies and percentages. Ordinal variables were described using distributions and, where appropriate, medians and interquartile ranges.

Associations between categorical variables were evaluated using Pearson’s chi-square test or Fisher’s exact test, as appropriate. Effect sizes were estimated using Cramér’s V. Non-parametric tests (Mann–Whitney U, Kruskal–Wallis) were used for ordinal data comparisons. Correlations were assessed using Spearman’s rank coefficient.

Internal consistency of selected item groupings was evaluated using Cronbach’s alpha, interpreted as part of an exploratory psychometric assessment rather than formal validation.

All tests were two-tailed, with statistical significance set at *p* < 0.05.

### 2.7. Exploratory Psychometric Evaluation

Given that the questionnaire was newly developed, an exploratory psychometric evaluation was conducted on selected item groupings. This analysis aimed to assess preliminary internal coherence rather than establish formal scale validation.

Only conceptually related items with appropriate response structures were included. Domains with limited variability or heterogeneous content were interpreted descriptively.

### 2.8. Home Monitoring Devices and Digital Applications

The use of home fetal monitoring devices in this study reflected real-world, non-standardized practice and was based entirely on participant self-report. The study did not aim to compare devices or evaluate their technical performance.

Participants most frequently reported the use of commercially available handheld Doppler devices, which provide intermittent fetal heart rate measurements. These devices are widely accessible consumer technologies intended primarily for personal use outside conventional clinical settings. Their performance may be influenced by gestational age, maternal characteristics, fetal position, and user experience.

Monitoring was performed independently by participants without standardized clinical supervision, and no clinical decisions were based on the recorded values within the scope of this study.

Many devices were used in conjunction with smartphone-based applications that enable visualization and storage of data. These applications function primarily as user interfaces and are not intended for clinical diagnosis or independent interpretation.

Accordingly, the findings of this study should be interpreted in the context of user-reported experience rather than device-specific clinical performance.

The present study focused on patient experience and perceptions related to device use rather than on the technical or clinical evaluation of the monitoring systems themselves.

### 2.9. Ethical Considerations

The study was conducted in accordance with the Declaration of Helsinki. Participation was voluntary and anonymous, and no identifiable personal data were collected.

Participants were informed about the study purpose and data use prior to questionnaire completion, and submission was considered to indicate informed consent.

The study was approved by the Ethics Committee of Filantropia Clinical Municipal Hospital (approval no. 22936 on 25 September 2024) and the Ethics Committee of the University of Medicine and Pharmacy of Craiova, Romania (approval no. 245 on 4 September 2024).

## 3. Results

### 3.1. Sociodemographic and Obstetric Characteristics of the Study Sample

A total of 225 women completed the questionnaire between August 2024 and March 2026. The largest age group was 31–35 years (*n* = 92, 40.9%), followed by 26–30 years (*n* = 66, 29.3%) and 36–40 years (*n* = 34, 15.1%). The mean number of pregnancies was 1.34 ± 0.75, and the mean number of live births was 0.97 ± 0.62. The detailed age distribution and obstetric profile of the participants are presented in Table 1.

**Figure 1:**
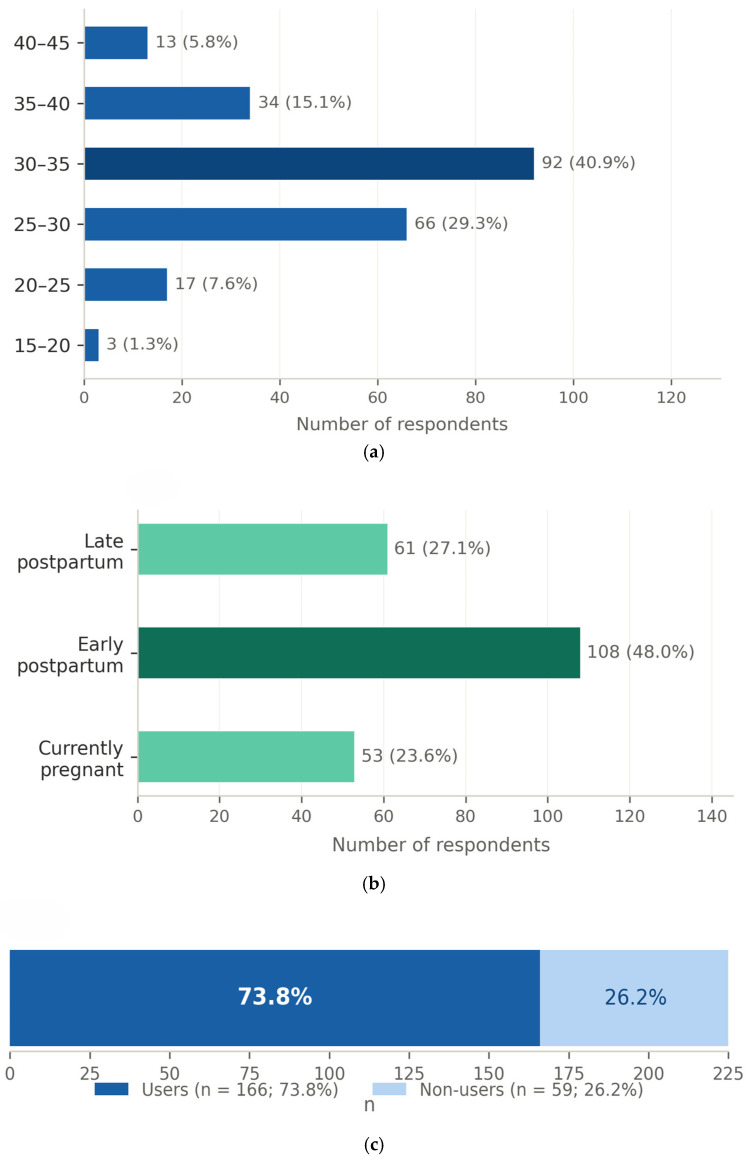
Sociodemographic characteristics and device adoption in the study sample (*n* = 225): (**a**) age-group distribution; (**b**) obstetric status at survey completion, categorized as currently pregnant, early postpartum (≤6 weeks after delivery), and later postpartum (>6 weeks after delivery); and (**c**) overall home fetal monitor use (users: *n* = 166, 73.8%; non-users: *n* = 59, 26.2%).

Regarding obstetric status at the time of survey completion, 53 women (23.6%) were currently pregnant, 108 women (48.0%) were in the early postpartum period (≤6 weeks after delivery), and 61 (27.1%) were in the later postpartum period (>6 weeks after delivery). The distribution of participants by obstetric status and device usage group is summarized in Table 1 and Figure 1.

### 3.2. Awareness and Adoption of Home Fetal Heart Rate Monitoring Devices

The majority of respondents (*n* = 197; 87.6%) reported prior awareness of home fetal monitoring devices. Overall, 166 women (73.8%) reported having used a home fetal monitoring device during pregnancy, forming the primary user group for subsequent analyses. The remaining 59 participants (26.2%) had never used such a device.

Among non-users, the most frequently reported reason for non-use was lack of awareness of the device (*n* = 34; 57.6%). Other reasons included unspecified concerns (*n* = 31; 52.5%), financial constraints (*n* = 7; 11.9%), lack of trust (*n* = 2; 3.4%), and perceived difficulty of use (*n* = 1; 1.7%). As multiple responses were allowed, percentages do not sum to 100%.

Among non-users who responded to this item (*n* = 59), the majority (50; 84.7%) indicated that the device would have been useful during pregnancy, highlighting a substantial perceived unmet need in this population.

A statistically significant association was identified between primiparous status and device adoption: primiparous women were significantly more likely to have used the device compared to multiparous women (χ^2^ = 24.445, df = 1, *p* < 0.001, Cramér’s V = 0.344), indicating a moderate effect size. Among primiparous respondents, 116 of 127 (91.3%) reported device use, compared to 49 of 79 multiparous women (62.0%).

### 3.3. Patterns of Device Use

Among device users, acquisition most commonly occurred between 25–30 and 30–35 weeks of gestation (*n* = 44 each; 23.9%), followed by 20–25 weeks (*n* = 31; 16.8%). More than half of users (*n* = 106; 52.7%) reported that earlier acquisition would have been preferable.

When asked to indicate the optimal timing for device acquisition, respondents most frequently selected 20–25 weeks (*n* = 41; 19.6%) and 25–30 weeks (*n* = 42; 20.1%), although a substantial proportion recommended earlier use, including 10–15 weeks (*n* = 35; 16.7%) and 15–20 weeks (*n* = 37; 17.7%). This discrepancy suggests a gap between actual acquisition timing and perceived optimal use, potentially reflecting delayed awareness or access to the device. Detailed distributions are presented in Table 2.

The most common pattern of use was once daily (*n* = 66; 34.7%), followed by twice daily (*n* = 39; 20.5%) and less frequent use (*n* = 37; 19.5%). Higher-frequency use was less common, with three times daily reported by 34 respondents (17.9%), four times daily by 3 (1.6%), and more than four times daily by 11 (5.8%). Nearly half of respondents (*n* = 97; 48.7%) reported using the device together with family members.

### 3.4. Sources of Information and Device Instruction

The attending gynecologist was the predominant source of device recommendation (*n* = 194; 93.7%), with only a small proportion of respondents reporting alternative sources such as the internet (*n* = 11; 5.3%), personal contacts (*n* = 1; 0.5%), or social media (*n* = 1; 0.5%).

**Figure 2:**
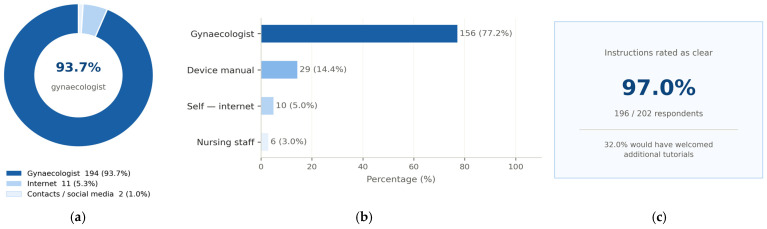
Sources of device recommendation and usage instruction among participants, with instruction clarity rating. (**a**) Source of device recommendation (*n* = 207); the attending gynecologist accounted for 93.7% of recommendations (*n* = 194), with internet (*n* = 11; 5.3%) and personal contacts or social media (*n* = 2; 1.0%) representing marginal sources. (**b**) Source of usage instructions (*n* = 202); the gynecologist explained device use to 77.2% of users (*n* = 156), followed by the device manual (*n* = 29; 14.4%), self-directed internet research (*n* = 10; 5.0%), and nursing staff (*n* = 6; 3.0%). (**c**) Instruction clarity rating; 97.0% of respondents (*n* = 196 of 202) found the provided instructions clear and understandable, although 32.0% indicated that additional tutorials or explanations would have been beneficial.

Similarly, device usage instructions were most frequently provided by the attending gynecologist (*n* = 156; 77.2%), followed by the device manual (*n* = 29; 14.4%), self-directed internet research (*n* = 10; 5.0%), nursing staff (*n* = 6; 3.0%), and personal contacts (*n* = 1; 0.5%). The distribution of information sources and instruction clarity is presented in Figure 2.

Most respondents rated the provided instructions as clear and understandable (*n* = 196; 97.0%). Nevertheless, 66 respondents (32.0%) indicated that additional explanations or online tutorials would have been beneficial, suggesting a residual educational need despite the overall high clarity of instructions.

### 3.5. Mobile Application Integration

Among respondents who provided data for this item (*n* = 197), 71 (36.0%) reported that their device was accompanied by a mobile application, while 126 (64.0%) indicated that no application was available. Among those who evaluated the application (*n* = 73), the most common perceptions were that it was essential (*n* = 30; 41.1%), as important as the monitor itself (*n* = 29; 39.7%), or that it provided a comprehensive overview of fetal status (*n* = 10; 13.7%), with only a minority reporting a neutral experience (*n* = 4; 5.5%).

Across respondents, 128 (63.7%) considered a mobile application to be mandatory for home fetal monitoring. A statistically significant association was observed between application availability and perceived necessity (χ^2^ = 15.260, df = 1, *p* < 0.001, Cramér’s V = 0.286), based on complete cases for both variables, indicating a moderate effect size. Respondents with application access were more likely to consider it mandatory (57 of 69; 82.6%) compared to those without access (62 of 117; 53.0%).

### 3.6. Emotional Experience and Perceived Reassurance During Device Use

**Figure 3:**
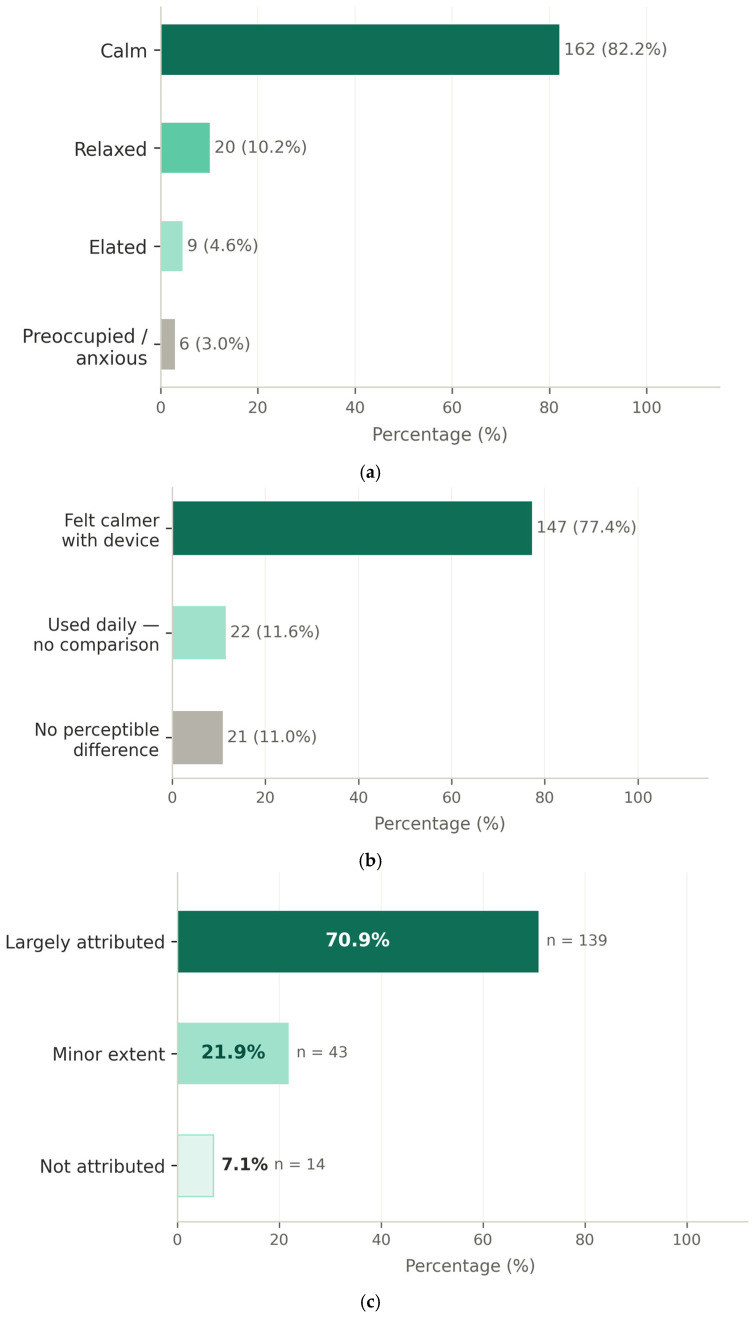
Emotional experience during device use and perceived contribution to maternal wellbeing among users (*n* = 166). (**a**) Emotional state reported during device use (*n* = 197); 82.2% of users reported feeling calm (*n* = 162), 10.2% relaxed (*n* = 20), and 4.6% elated (*n* = 9), yielding an overall positive emotional response rate of 92.4%; 3.0% reported feeling preoccupied or anxious (*n* = 6). (**b**) Subjective experience on days with versus without device use (*n* = 190); 77.4% of users reported feeling calmer on days when the device was used (*n* = 147), 11.6% used the device daily without interruption (*n* = 22), and 11.0% perceived no difference (*n* = 21). (**c**) Perceived contribution of device use to maternal wellbeing (*n* = 196); 70.9% of respondents largely attributed their sense of wellbeing to device use (*n* = 139), 21.9% to a minor extent (*n* = 43), and 7.1% reported no attributed contribution (*n* = 14).

Among respondents who answered this item (*n* = 197), the most frequently reported emotional state during device use was feeling calm (*n* = 162; 82.2%), followed by relaxed (*n* = 20; 10.2%), elated (*n* = 9; 4.6%), and preoccupied or anxious (*n* = 6; 3.0%). Overall, 92.4% of device users reported a positive emotional response during monitoring (Figure 3).

When comparing days of device use with days without monitoring (*n* = 190), 147 respondents (77.4%) reported feeling calmer on days when they used the device; 22 (11.6%) reported no variation as they used the device daily without interruption; and 21 (11.0%) reported no perceptible difference.

Regarding perceived reassurance associated with device use (Q28), 139 respondents (70.9%) attributed their overall sense of wellbeing largely to device use, 43 (21.9%) to a minor extent, and 14 (7.1%) did not identify a contribution. The internal consistency of the emotional impact subscale (comprising perceived emotional state, maternal wellbeing attribution, daily experience difference, and neonatal wellbeing attribution) was modest but acceptable for exploratory use (Cronbach’s α = 0.639, *n* = 183, k = 4 items).

An additional rating item (Q34) asked participants to score the contribution of device use to their wellbeing on a 1–5 scale; however, this item exhibited a markedly bimodal distribution (score 1: *n* = 49; score 5: *n* = 55) and was not significantly correlated with the categorical wellbeing item (Spearman’s ρ = −0.075, *p* = 0.303), suggesting potential variability in the interpretation of scale anchors by respondents. This item was therefore excluded from subscale analyses and is reported descriptively only (mean ± SD: 3.10 ± 1.54, median = 3; *n* = 191).

### 3.7. User-Reported Reassurance and Safety Perceptions

Regarding perceived reassurance regarding fetal status, 101 of 193 respondents (52.3%) reported increased reassurance regarding fetal status during device use, while 92 (47.7%) did not. A fetal monitoring finding that prompted additional medical evaluation was reported by 15 respondents (7.6%).

Among respondents who answered these items, no participants reported perceived maternal harm (*n* = 197; 100%) or neonatal harm (*n* = 195; 100%) associated with device use. These findings reflect a favorable safety perception among users; however, no objective clinical safety outcomes were assessed.

### 3.8. Attitudes, Recommendations, and Overall Evaluation

Most respondents recommended the use of home fetal monitors to all pregnant women (*n* = 192; 94.1%). More than half of respondents (*n* = 122; 59.2%) expressed the opinion that home fetal monitoring devices should be routinely used during pregnancy. Given the exploratory nature of the survey, this finding should be interpreted as a reflection of participant attitudes rather than as evidence supporting policy recommendations or clinical requirements. In terms of overall appraisal, 168 respondents (82.0%) reported only advantages, while 37 (18.0%) acknowledged both advantages and disadvantages.

A statistically significant association was observed between primiparous status and overall evaluation: primiparous women were more likely to report an exclusively positive appraisal (χ^2^ = 8.721, df = 1, *p* = 0.003, Cramér’s V = 0.212). The composite positive perception subscale across six evaluative items demonstrated modest internal consistency (Cronbach’s α = 0.664, *n* = 180, k = 6 items).

Among multiparous women who had not used the device in a prior pregnancy but used it subsequently, responses were dichotomous, with 54.8% reporting observable differences between pregnancies. Open-ended responses, although not formally analyzed using qualitative methods, most frequently emphasized reassurance, peace of mind, emotional comfort, and increased perceived connection with the fetus as perceived benefits of device use. Reported disadvantages were relatively infrequent and generally related to occasional anxiety, uncertainty regarding interpretation, or concerns about overuse. A summary of attitudinal outcomes is presented in Table 3.

### 3.9. Internal Consistency of Preliminary Subscales

Given the exploratory nature of this instrument, a preliminary assessment of internal consistency was performed for conceptually defined item groupings (Table 4).

The emotional impact subscale (S2; Q25, Q28, Q36, Q27) yielded a Cronbach’s α of 0.639 (*n* = 183), while the broader composite positive perception subscale (S5; six evaluative items) yielded α = 0.664 (*n* = 180). These values indicate acceptable internal consistency for a pilot instrument, approaching the conventional threshold of α ≥ 0.70.

In contrast, the attitudinal subscale (S1), composed of recommendation behavior (Q16), support for mandatory use (Q29), and overall evaluation of the device (Q37), demonstrated lower internal consistency (Cronbach’s α = 0.501), likely reflecting conceptual heterogeneity within the construct. In particular, the item assessing mandatory use (Q29) may capture a more normative or policy-oriented perspective rather than a purely experiential attitude toward device use. These findings should therefore be interpreted cautiously within the exploratory framework of the present study. Overall, these findings suggest that the instrument demonstrates preliminary structural coherence, while also identifying specific domains that require refinement and re-specification in future validation studies. Additional item-level analyses showed that corrected item–total correlations were generally acceptable for the exploratory context of the study, although some items displayed weaker associations with their respective subscales. Cronbach’s alpha if item deleted analyses suggested that the item assessing mandatory use contributed least to the internal consistency of the attitudinal subscale, supporting the interpretation that it may reflect a partially distinct normative dimension. Across the broader composite subscale, removal of individual items did not lead to substantial improvement in internal consistency, supporting retention of the current structure for exploratory purposes.

### 3.10. Summary of Inferential Findings

Three independent statistically significant associations were identified through chi-square analysis (Table 5). First, primiparous women demonstrated significantly higher rates of device adoption (Cramér’s V = 0.344), representing the strongest association observed in this study. Second, women with access to a mobile application were significantly more likely to consider it mandatory (V = 0.286). Third, primiparous women were more likely to report an exclusively positive overall evaluation of the device (V = 0.212). No additional associations reached statistical significance at the predefined threshold (α = 0.05).

## 4. Discussion

### 4.1. Home Fetal Monitoring in Contemporary Prenatal Care

Although pregnancy is generally considered a normal physiological state, it continues to be associated with clinically relevant maternal and fetal risks [8,15]. Continuous surveillance is therefore essential; however, the evolution of monitoring technologies in obstetrics has been relatively modest. In routine practice, fetal assessment is still predominantly based on cardiotocography, which requires abdominal sensors connected to fixed monitoring systems. Such setups may limit maternal movement and confine monitoring to hospital environments. Moreover, the evaluation of maternal physiological parameters is often performed using separate devices and at discrete time points, which may reduce the overall coherence and continuity of clinical monitoring, particularly in the early identification of pregnancy-related complications [2,8].

The growing adoption of remote fetal heart rate (FHR) monitoring has introduced new possibilities for assessing fetal well-being beyond conventional clinical settings. By removing spatial and temporal constraints, this approach enables more flexible and continuous forms of prenatal surveillance. Its clinical relevance became particularly apparent during the COVID-19 pandemic, when remote monitoring reduced the need for in-person visits and limited potential exposure risks. Beyond this context, such technologies have demonstrated considerable potential for long-term integration into routine prenatal care pathways, with increasing interest in their potential to support patient engagement and timely communication with healthcare professionals [3].

Although home fetal heart rate monitoring devices are generally considered safe, non-invasive technologies, several safety-related considerations should be acknowledged. Potential limitations include variable signal quality, user-dependent differences in device operation, and the possibility of inaccurate recordings or interpretation errors. In addition, apparently reassuring findings may create false reassurance and potentially delay medical consultation when maternal symptoms or concerns arise. Therefore, home monitoring devices should not be regarded as substitutes for routine prenatal care or professional fetal assessment, but rather as complementary tools that may support patient engagement and reassurance when used within supervised prenatal care pathways and accompanied by appropriate patient education.

In this context, the present cross-sectional pilot study assessed awareness, adoption, user experience, emotional impact, and overall attitudes toward home fetal heart rate monitoring devices among pregnant and postpartum women in Romania. The adoption rate observed in our cohort (73.8%) is consistent with previously reported levels of acceptability for remote fetal monitoring technologies. Supporting this observation, a systematic review and meta-analysis by Li et al. (*n* = 1128) demonstrated that remote fetal monitoring was associated with improved neonatal outcomes, including a reduced risk of neonatal asphyxia (RR 0.66; 95% CI 0.45–0.97), as well as lower healthcare costs [16]. Similarly, Crawford et al. reported high acceptability rates (90–100%) for home cardiotocography and ultrasound in high-risk pregnancies, with 73% of participants recommending continued use after study completion [17]. These findings provide a broader clinical context, while the present study specifically adds patient-reported evidence regarding acceptability and real-world engagement with home monitoring.

### 4.2. Patient Acceptance and the Role of Healthcare Professionals

Notably, the proportion of participants in our study who would recommend the device to all pregnant women (94.1%) was particularly high. This finding suggests that real-world user satisfaction may exceed that observed in controlled study settings. Such differences may reflect increased perceived autonomy, ease of use, and the psychological reassurance provided by on-demand fetal monitoring, which may also influence the timeliness with which patients seek medical evaluation when concerns arise. These results indicate that patient acceptance is influenced not only by usability, but also by the perceived enhancement of control and emotional security during pregnancy. Beyond usability and clinical performance, home monitoring technologies also reshape the patient–clinician relationship by extending surveillance into the home environment and increasing patient involvement in care processes [12,14].

The attending gynecologist emerged as the primary source of both device recommendation (93.7%) and usage instruction (77.2%), highlighting the central role of clinicians in facilitating the adoption of digital monitoring technologies. This observation is consistent with previous studies showing that pregnant women place greater trust in clinician-endorsed information compared to informal or digital sources. For example, Matvienko-Sikar et al. reported higher confidence in health technologies recommended by healthcare providers [18], while Pontones et al. demonstrated that supervised use of mobile ultrasound was associated with high acceptability and a preference for real-time professional guidance [19]. From a clinical perspective, this reinforces that the potential value of home monitoring lies not only in patient experience but also in its integration within supervised care pathways where user concerns and device-recorded findings can be appropriately discussed and contextualized.

Overall, these findings suggest that the successful implementation of home fetal monitoring technologies depends not only on device performance but also on active clinician involvement. Integrating such technologies into routine prenatal care therefore requires not only technical optimization but also targeted educational strategies for healthcare providers to support appropriate use and patient guidance.

### 4.3. Adoption Patterns and Primiparity as a Driver of Technology Update

In recent years, remote monitoring approaches have become increasingly relevant in the management of high-risk pregnancies, offering an alternative to traditional models of care that rely on frequent hospital visits. These strategies enable ongoing clinical supervision while allowing patients to remain in their home environment, thereby reducing the need for continuous in-person attendance, while maintaining continuous communication and follow-up within prenatal care pathways [9,20].

In a broader sense, telemonitoring encompasses the use of digital health technologies—including mobile platforms, wireless devices, and communication systems—to collect, transmit, and evaluate clinical data. This approach facilitates continuous interaction between patients and healthcare providers and supports timely clinical decision-making [13,14,20]. The most robust inferential finding of this study was the significantly higher rate of device adoption among primiparous women compared with multiparous women (χ^2^ = 24.445, df = 1, *p* < 0.001, Cramér’s V = 0.344), representing a medium-magnitude effect. This result aligns with a substantial body of literature indicating that primiparous women experience higher levels of pregnancy-related uncertainty, anxiety, and perceived vulnerability [21,22].

Recent developments in wireless sensing and mobile technologies have made it possible to design integrated systems that can record both fetal and maternal physiological signals and transmit them in real time to mobile devices [7]. At the same time, obtaining a clear signal remains difficult, as non-invasive recordings are often affected by maternal cardiac activity, respiration, movement artifacts, and environmental noise, which can reduce the accuracy and reliability of fetal heart rate measurements, and may affect signal quality and interpretation reliability in non-clinical settings [6,23].

Previous research has consistently demonstrated that first-time mothers report greater fear of childbirth and lower self-efficacy. For example, Shakarami et al. found significantly higher Delivery Fear Scale scores among primiparous women, alongside reduced confidence in childbirth, while large prospective cohorts have confirmed the association between nulliparity and increased pregnancy-specific anxiety [24,25]. In this context, the increased adoption of home monitoring observed in our study may be interpreted as a behavioral response to heightened informational and emotional needs.

This interpretation is further supported by findings from Gan et al., who reported high acceptability of remote fetal heart rate self-monitoring among nulliparous women and attributed this to a “fear of the unknown” driving the search for reassurance [22]. Similarly, Wang et al., using a Technology Acceptance Model framework, identified health anxiety as a significant predictor of perceived usefulness and intention to adopt smart fetal monitoring devices [26].

In this context, these findings suggest that the adoption of home fetal monitoring among primiparous women is not merely a function of access or recommendation, but reflects a broader adaptive strategy aimed at reducing uncertainty and enhancing perceived control during pregnancy. This behavioral dimension may represent a key target for future interventions seeking to optimize the integration of digital monitoring tools in prenatal care.

### 4.4. Emotional Reassurance and Perceived Well-Being

The emotional impact of home fetal monitoring represents one of the most relevant patient-reported dimensions of the present study. A substantial majority of users reported a positive emotional experience during device use (92.4%), with calmness identified as the predominant state (82.2%). Moreover, over three-quarters of respondents (77.4%) reported feeling calmer on days when the device was used compared with days without monitoring, indicating a consistent within-subject perception of emotional benefit. Similar patient-centered findings have also been reported in recent telemonitoring feasibility studies across different pregnancy risk group [27].

These findings support the interpretation that accessible, on-demand fetal heartbeat detection may act as a form of emotional self-regulation during pregnancy, reducing uncertainty and reinforcing perceived maternal–fetal connection [4]. However, this reassurance may be clinically beneficial only when it does not delay appropriate medical consultation in the presence of abnormal findings. This aligns with qualitative insights reported by Wakefield et al., who identified reassurance, reduced anxiety, and convenience as central themes in user experiences with wearable fetal ECG monitoring [28].

Further support for this mechanism is provided by interventional evidence. In a randomized controlled trial (RCT), Mor et al. demonstrated that home ultrasound monitoring significantly reduced maternal anxiety among women with a history of recurrent pregnancy loss compared with standard care [29]. Although derived from a different clinical population, these findings lend experimental support to the reassurance-based pathway suggested by our observational data, reinforcing the role of home monitoring technologies as potential tools for psychological support in prenatal care.

The observation that 92.8% of users attributed their overall sense of well-being wholly or partially to device use (70.9% and 21.9%, respectively) warrants careful interpretation. As a self-reported measure derived from cross-sectional survey data, this finding is inherently susceptible to attribution bias and cannot support causal inference regarding the effect of device use on maternal well-being.

Nevertheless, the consistency of this perception with prior evidence on the psychosocial impact of prenatal monitoring technologies suggests that it may represent a meaningful subjective outcome. In a narrative review of telehealth interventions in antenatal care, Atkinson et al. reported that patient-perceived benefits of remote monitoring consistently include reassurance, enhanced sense of control, and increased engagement with care processes [30].

Within this framework, the high level of well-being attribution observed in our study may be interpreted as reflecting a perceived benefit mediated through psychological and behavioral pathways, rather than a direct causal effect. This distinction is particularly relevant when considering the integration of such technologies into routine prenatal care, where subjective experience and perceived reassurance may play a complementary role alongside clinical outcomes. Such perceived benefits may influence healthcare-seeking behavior, potentially affecting the timing of presentation in cases of suspected complications.

### 4.5. Timing of Adoption and User Expectations

The gestational age distribution of device acquisition, with peak uptake occurring at 25–30 and 30–35 weeks of gestation, indicates that adoption predominantly takes place during the late second and third trimesters. This timing corresponds to both the onset of consistent fetal movement perception and the increasing clinical emphasis on fetal surveillance as pregnancy advances.

However, a notable discrepancy emerges between observed behavior and perceived optimal timing. More than half of users (52.7%) reported that earlier acquisition would have been beneficial, with the most frequently recommended windows centered around 20–25 and 25–30 weeks, and a substantial proportion (16.7%) favoring adoption as early as 10–15 weeks. This divergence suggests that device uptake is not solely determined by perceived utility but is likely influenced by external constraints such as delayed awareness, financial considerations, or the timing of clinician recommendations.

From an adoption perspective, this pattern may reflect a form of delayed uptake, whereby intention precedes action but is mediated by contextual barriers. Supporting this interpretation, Tamaru et al. demonstrated the feasibility of mobile cardiotocography across the second half of pregnancy in low-risk populations, indicating that earlier use is technically viable [14,31]. Taken together, these findings highlight a potential window for earlier intervention and suggest that optimizing the timing of information delivery and recommendation could facilitate a more timely adoption of home fetal monitoring technologies.

### 4.6. Digital Integration and Future Monitoring Ecosystems

The observed association between mobile application availability and the perceived necessity of such functionality (χ^2^ = 15.260, df = 1, *p* < 0.001, Cramér’s V = 0.286) highlights the importance of experiential factors in shaping technology evaluation. Among women whose device included an application, 82.6% considered it mandatory, compared with 53.0% of those without access, suggesting that the perceived value of digital integration is reinforced through direct use rather than anticipated a priori.

This finding also reveals a notable discrepancy between current device characteristics and user expectations. While only 36.0% of devices in the present sample were accompanied by a mobile application, nearly two-thirds of respondents (63.7%) considered such integration essential. This mismatch points to an evolving standard in user expectations, whereby standalone monitoring devices are increasingly perceived as insufficient in the absence of complementary digital functionalities.

Although cardiotocography remains widely used in clinical practice, its interpretation is inherently limited by a degree of subjectivity. Variability in clinical judgment persists even among experienced practitioners, especially when assessing heart rate patterns and classifying tracings. Moreover, while abnormal patterns may be associated with fetal hypoxia, their ability to accurately predict adverse neonatal outcomes is limited. As a result, cardiotocography is better regarded as a screening tool with high sensitivity but relatively low specificity, highlighting the need for complementary approaches that may support earlier detection of fetal compromise outside traditional clinical settings [5,10,32].

Taken together, these limitations emphasize the need for more objective and reliable monitoring approaches that can improve diagnostic accuracy and support earlier identification of fetal compromise.

These observations are consistent with broader developments in digital health, where the integration of hardware, software, and connectivity into unified monitoring ecosystems has become central to patient engagement and sustained use. Atkinson et al. reported that telehealth solutions combining device-based monitoring with data interpretation and communication tools are associated with higher levels of patient engagement and adherence [30]. Similarly, Eswaran et al., in a scoping review of digital technologies in antenatal care, identified mobile application integration as a rapidly expanding domain, with increasing convergence between monitoring devices and digital platforms since 2019 [33].

### 4.7. Safety Considerations and Clinical Implications

Within this context, the findings of the present study suggest that the future acceptability and effectiveness of home fetal monitoring technologies may depend not only on the performance of the device itself, but also on the extent to which it is embedded within an integrated digital ecosystem that supports interpretation, feedback, and communication, which may be essential for translating home monitoring data into clinically actionable information in the context of pregnancy complications [1].

Safety perceptions reported in the present study require careful contextual interpretation. In this study, safety considerations refer to perceived safety as reported by participants rather than objectively measured clinical safety outcomes. No respondent reported perceived harm to either herself or her newborn (100%), and a relatively small proportion of users (7.6%) reported experiencing a monitoring finding that prompted additional medical evaluation during device use. While these findings are reassuring, they must be interpreted within the constraints of a self-reported, cross-sectional design, which does not permit causal inference or objective assessment of adverse outcomes.

Nevertheless, the absence of reported harm in this cohort is consistent with existing evidence on consumer-grade fetal monitoring technologies. Porter et al., in a prospective accuracy study, demonstrated that a wireless, self-guided fetal heartbeat monitor showed strong agreement with standard cardiotocography and could be safely self-administered at home, with no device-related adverse events reported [34]. Similarly, Kariman et al., in a recent systematic review of tele-ultrasound and handheld devices in pregnancy, concluded that safety profiles across included studies were generally favorable, with identified risks primarily related to misinterpretation of findings rather than intrinsic device-related harm [35].

The identification of abnormal heart rate patterns allows clinicians to recognize early signs of fetal compromise and to initiate timely obstetric interventions. In clinical practice, particularly in late pregnancy, the use of these monitoring systems has been associated with improved fetal surveillance and a lower incidence of adverse perinatal outcomes [36,37].

This distinction is clinically relevant, as it suggests that the principal safety considerations associated with home fetal monitoring may lie not in the physical use of the device itself, but also in its potential as a complementary monitoring approach that could prompt earlier clinical assessment when abnormalities are suspected. Accordingly, appropriate user education and clinician guidance remain essential to ensure safe and effective integration of such technologies into prenatal care.

The observed alert rate of 7.6% warrants further consideration from a clinical perspective, as it may reflect instances in which home monitoring identified potential early signs of fetal compromise. This subgroup of users who received a device-generated signal suggestive of potential fetal compromise may represent instances in which home monitoring functioned as an early warning or triage mechanism, potentially prompting timely clinical evaluation. If validated, such signals could position home monitoring devices as adjunct tools for early triage, particularly in settings with limited access to continuous clinical surveillance.

However, in the absence of linked clinical outcome data, the diagnostic performance of these alerts cannot be established. In particular, key parameters such as positive predictive value, false-positive rates, and subsequent clinical actions remain unknown.

This limitation highlights an important gap in the current evidence base, namely the lack of integrated studies combining user-reported data with verified clinical outcomes. Addressing this gap will be essential for determining whether home fetal monitoring devices can move beyond a reassurance-focused role toward a validated role in supporting early detection and triage of pregnancy complications within prenatal care pathways.

### 4.8. Study Limitations

This study has several limitations. First, the cross-sectional design, reliance on self-reported data, and use of convenience sampling could have introduced recall and response biases and restricted the generalizability of the findings. Furthermore, the questionnaire was developed specifically for this exploratory investigation and, although preliminary assessments of internal consistency were conducted, it was not formally validated using standardized psychometric procedures.

Second, the study focused on subjective perceptions, attitudes, and experiences related to home fetal heart rate monitoring and did not assess objective clinical outcomes, diagnostic performance, safety endpoints, or healthcare utilization measures.

Third, the monitoring devices used by participants differed in their technical specifications and usage patterns, and qualitative responses were analyzed descriptively rather than through established qualitative research methodologies. Although these characteristics reflect real-world practice, they limit device-specific conclusions and reduce the depth of qualitative interpretation.

Despite these limitations, this study provides real-world evidence regarding awareness, acceptance, and perceived reassurance associated with home fetal heart rate monitoring. Future prospective studies using validated instruments, standardized monitoring protocols, and clinical outcome measures are needed to further clarify the role of these technologies in prenatal care.

### 4.9. Future Directions

Future research in this field should focus on three key areas. First, formal psychometric validation of the questionnaire instrument is required, including assessment of test–retest reliability, factor structure, and cross-cultural applicability. Second, prospective cohort studies and randomized controlled trials linking device use with objective maternal and fetal outcomes are needed to determine clinical effectiveness and cost-effectiveness. Third, qualitative and mixed-methods research exploring healthcare provider perspectives—particularly regarding their readiness to recommend and support home monitoring technologies—would provide valuable complementary insights to the patient-centered findings reported here.

## 5. Conclusions

This study shows that home fetal heart rate monitoring is highly acceptable among pregnant and postpartum women, with substantial adoption and strong perceived emotional benefit in a real-world setting. Device use appears to fulfill an important psychological and informational function, particularly among primiparous women, by reducing uncertainty and enhancing perceived control during pregnancy. Adoption patterns were strongly influenced by clinician involvement and by the availability of integrated digital functionalities, highlighting the importance of professional guidance and user-centered technology design. Overall, these findings support the potential role of home fetal monitoring as a complementary component of patient-centered digital prenatal care. However, in the absence of linked clinical outcome data, its clinical utility and optimal integration into supervised prenatal care pathways require further prospective evaluation.

## Figures and Tables

**Table 1:** Sociodemographic and obstetric characteristics of the study sample (*n* = 225).

Characteristic	Total Sample *n* = 225 (%)	Users *n* = 166 (%)	Non-Users *n* = 59 (%)
**Age group (years)**			
18–20	3 (1.3)	3 (1.8)	0 (0.0)
21–25	17 (7.6)	11 (6.6)	6 (10.2)
26–30	66 (29.3)	46 (27.7)	20 (33.9)
31–35	92 (40.9)	72 (43.4)	20 (33.9)
36–40	34 (15.1)	26 (15.7)	8 (13.6)
41–45	13 (5.8)	8 (4.8)	5 (8.5)
**Obstetric status at survey completion**			
Currently pregnant	53 (23.6)	41 (24.7)	12 (20.3)
Early postpartum (≤6 weeks)	108 (48.0)	86 (51.8)	22 (37.3)
Late postpartum (>6 weeks)	61 (27.1)	38 (22.9)	23 (39.0)
Not reported	3 (1.3)	1 (0.6)	2 (3.4)
**Parity at the time of device use (respondents with available data, *n* = 206)**			
Primiparous	127 (61.7)	–	–
Multiparous	79 (38.3)	–	–
Number of pregnancies, mean ± SD	1.34 ± 0.75 *	–	–
Number of live births, mean ± SD	0.97 ± 0.62 *	–	–

* SD: standard deviation; –: variable not stratified for this subgroup (Note: Not all questions were answered by all participants; therefore, the number of valid responses differs across variables).

**Table 2:** Device usage patterns among users (*n* = 166) and recommended gestational age for acquisition (*n* = 209).

Variable	*n*	%
*Gestational age at device acquisition (n = 184)*		
5–10 weeks	5	2.7
10–15 weeks	25	13.6
15–20 weeks	19	10.3
20–25 weeks	31	16.8
25–30 weeks	44	23.9
30–35 weeks	44	23.9
35–40 weeks	16	8.7
*Would have been useful to acquire earlier (n = 201)*		
Yes	106	52.7
No	95	47.3
*Recommended gestational age for acquisition (n = 209)*		
5–10 weeks	15	7.2
10–15 weeks	35	16.7
15–20 weeks	37	17.7
20–25 weeks	41	19.6
25–30 weeks	42	20.1
30–35 weeks	29	13.9
35–40 weeks	10	4.8
*Daily frequency of use (n = 190)*		
Rarely/less than once daily	37	19.5
Once daily	66	34.7
Twice daily	39	20.5
Three times daily	34	17.9
Four times daily	3	1.6
More than four times daily	11	5.8

Percentages are calculated based on the number of respondents who provided valid answers to each item.

**Table 3:** Attitudinal outcomes and overall evaluation of home fetal monitoring (users, *n* = 166).

Variable	*n*	% *
*Recommends device to all pregnant women (n = 204)*		
Yes	192	94.1
No	12	5.9
*Considers use should be mandatory (n = 206)*		
Yes	122	59.2
No	84	40.8
*Overall evaluation (n = 205)*		
Advantages only	168	82.0
Both advantages and disadvantages	37	18.0
*Monitoring finding prompting additional medical evaluation (n = 198)*		
Yes	15	7.6
No	183	92.4

* Percentages calculated from the number of respondents providing valid answers to each item (Note: Not all questions were answered by all participants; therefore, the number of valid responses differs across variables).

**Table 4:** Preliminary internal consistency of conceptual subscales.

Subscale	Items Included	k	*n*	Cronbach’s α
S1—Attitudinal evaluation	Q16, Q29, Q37	3	194	0.501
S2—Emotional impact	Q25, Q28, Q36, Q27	4	183	0.639
S5—Broad composite	Q25, Q36, Q28, Q27, Q16, Q37	6	180	0.664

k: number of items; *n*: number of respondents with complete data. Q34 was excluded from subscale analyses due to its bimodal distribution and lack of correlation with the corresponding categorical item (Spearman’s ρ = −0.075, *p* = 0.303). Conventional threshold for acceptable internal consistency is α ≥ 0.70; values ≥ 0.60 are considered acceptable in exploratory or pilot studies.

**Table 5:** Summary of statistically significant associations identified by chi-square analysis.

Association	χ^2^	df	*p*-Value	Cramér’s V	Effect Size
Device adoption ~ Primiparous status	24.445	1	<0.001	0.344	Medium
App mandatory ~ App available	15.260	1	<0.001	0.286	Small–medium
Advantages only ~ Primiparous status	8.721	1	0.003	0.212	Small–medium

Effect size interpretation for Cramér’s V (2 × 2 tables): small ≈ 0.10, medium ≈ 0.30, large ≈ 0.50.

## Data Availability

The datasets presented in this article are not readily available due to the need to uphold intellectual property rights and confidentiality agreements, ensure the integrity and accuracy of ongoing analyses, and comply with ethical and regulatory guidelines that govern data dissemination before formal publication. Requests to access the datasets should be directed to the authors (sidonia.sandulescu@umfcv.ro and anca.vulcanescu@umfcv.ro).

## Abbreviations

The following abbreviations are used in this manuscript:

CI	Confidence interval
CTG	Cardiotocography
df	Degrees of freedom
FHR	Fetal heart rate
*n*	Number of respondents
RCT	Randomized controlled trial
RR	Risk ratio
SD	Standard deviation
SPSS	Statistical Package for the Social Sciences
α	Cronbach’s alpha (internal consistency coefficient)
χ^2^	Chi-square statistic
ρ	Spearman’s rank correlation coefficient
V	Cramér’s V (effect size measure)
